# Landscape of m^6^A RNA methylation regulators in liver cancer and its therapeutic implications

**DOI:** 10.3389/fphar.2024.1376005

**Published:** 2024-03-13

**Authors:** Jindu Zhao, Guo-Ying Li, Xian-Ying Lu, Li-Ran Zhu, Qun Gao

**Affiliations:** ^1^ Department of Oncology Surgery, Anhui Medical University Children’s Medical Center, Anhui Provincial Children’s Hospital, Hefei, Anhui, China; ^2^ College of Integrative Medicine, Anhui University of Chinese Medicine, Hefei, Anhui, China; ^3^ Anhui Institute of Pediatric Research, Anhui Medical University Children’s Medical Center, Anhui Provincial Children’s Hospital, Hefei, Anhui, China

**Keywords:** liver cancer, N6-methyladenosine (m^6^A) modification, HCC, ICC, HB

## Abstract

Liver cancer remains as the third leading cause of cancer-related death globally as of 2020. Despite the significant progress made in the field of liver cancer treatment, there is still a lack of effective therapies in patients with advanced cancer and the molecular mechanisms underlying liver cancer progression remain largely elusive. N6-methyladenosine (m^6^A) modification, as the most prevalent and abundant internal RNA modification in eukaryotic RNAs, plays an essential role in regulating RNA metabolism including RNA splicing, stability, translation, degradation. To date, there is mounting evidence showing that m^6^A dysregulation is closely associated with the onset and development of many tumors including hepatocellular carcinoma (HCC), intrahepatic cholangiocarcinoma (ICC) and hepatoblastoma (HB). In this review, we summarize the last research progress regarding the functions of m^6^A-related regulators in liver cancer and its underlying mechanisms. Additionally, we also discuss the therapeutic applications of m^6^A-based inhibitors in liver cancer treatment.

## 1 Introduction

Liver cancer, a disease starting in the cells lining in the liver tissues, represents as one of the most life-threatening events worldwide (Llovet et al., 2016). Also, liver cancer remains as the third leading cause of cancer-related death globally as of 2020, with an estimated 830,000 individuals died from this disease in 2020 (Sung et al., 2021). A literature published in JAMA Oncology suggested that the number of diagnosed cases of liver cancer increased by 75% globally between 1990 and 2015 (Akinyemiju et al., 2017). Moreover, it has been estimated that, by 2025, more than 1 million individuals will be diagnosed with liver cancer annually (Bray et al., 2018). Despite the significant progress made in the field of liver cancer treatment, there is still lacking of effective therapies in advanced patients and the molecular mechanisms underlying liver cancer progression remain largely elusive, thereby warranting a more in-depth exploration (Vogel et al., 2022). Of note, a large body of research documents a high connectivity of epigenetic dysregulation and cancer development including HCC (Cheng Y. et al., 2019). To date, owing to a fast and wide application of next-generation sequencing (NGS) in epigenetic studies, reversible RNA modifications such as N1-methyladenosine (m^1^A), 5-methylcytosine (m^5^C), N6-methyladenosine (m^6^A) as well as N7-methylguanosine (m^7^G) emerge as critical players of posttranscriptional gene expression, thus exerting remarkably key roles in regulating diverse cellular processes (Shi H. et al., 2020). Among these modifications, m^6^A modification has attracted great attention in the past years as a result of its significance in regulating gene expression and dictating cell fate (Ji and Chen, 2012; Hori, 2014). Moreover, there is plenty of evidence linking dysregulated m^6^A modification with tumorigenesis, including liver cancer (Gao et al., 2021). Consequently, a continuous exploration of the roles and molecular mechanisms of m^6^A modification may facilitate the development of novel therapeutic approaches against liver cancer. Herein, our paper aimed to summarize the recent advancements related to the functions of m^6^A-related regulators in liver cancer and its associated mechanisms, thus in hope of offering new insights into the potential implications of m^6^A modification in the diagnosis and treatment of liver cancer.

## 2 Liver carcinogenesis

Liver cancer represents as one of the fastest growing cancer types globally, accounting for 4.7% of the total cancer cases in 2020, and its incidence has gradually increasing in the past decades (Sung et al., 2021). HCC, a type of cancer arising from hepatocytes, constitutes nearly 85% of primary liver cancer. Generally, HCC patients are known to have an unfavorable prognosis, with a 5-year survival rate of 20%–40%. Moreover, patients with advanced HCC show worse clinical outcomes (Yang et al., 2019). ICC, a type of primary liver malignancy that originated from the intrahepatic bile ducts, comprises of 10%–15% of all primary liver malignant tumor. Besides, patients with ICC have the worst prognosis of any tumor arising in the liver, with a five-year overall survival of nearly 9% and a high recurrence rate (Blechacz et al., 2011).

Currently, there is evidence of viral hepatitis, high alcohol consumption, smoking, obesity as well as non-alcoholic fatty liver disease (NAFLD) as risk factors for HCC (El-Serag, 2012; Estes et al., 2018). Of note, chronic hepatitis B virus (HBV) infection is well-recognized as the leading cause of HCC globally, particularly in eastern Asia and Sub-Saharan Africa while hepatitis C virus (HCV) is the major risk factor together with heavy drinking in Western and Japan (Global Burden of Disease Liver Cancer Collaboration et al., 2017). Recently, growing evidence suggests that NAFLD is becoming the fastest growing contributor of HCC worldwide, especially in the USA (Estes et al., 2018). Despite that risk factors for ICC remains elusive, several lines of evidence reveal infectious causes such as liver fluke infection, primary sclerosing cholangitis (PSC), and hepatolithiasis can increase the risk of developing ICC (Bagante et al., 2016).

Currently, our understanding of the pathophysiology of HCC explains only a small part of the big picture as HCC pathophysiology is a complex and multiple process that involves the interplay between a variety of factors including cellular microenvironment, gene mutations, epigenetic modification, and so on (Dhanasekaran et al., 2016). Also, malignant transformation of hepatocytes may arise from a sequence of multiple genomic mutations in cancer driver genes (e.g., TP53, RB1, CCNE1, PTEN and AXIN1), activation of several signal pathways such as Wnt/β-catenin and insulin/IGF-1/IRS-1/MAPK, altered microenvironment, and epigenetic variation, which can explain the high heterogeneity of HCC (Thompson and Monga, 2007; Whittaker et al., 2010; Miamen et al., 2012).

Considerable advances have been made over the past decades in the field of HCC treatment. To date, treatment options for HCC include hepatic resection, liver transplantation, alation, embolization, and systemic therapies (Llovet et al., 2016). Barcelona Clinic Liver Cancer (BCLC) staging system, as the most often utilized HCC staging system, can provide specific therapeutic options, depending on the extent of tumor burden, severity of liver function, and performance status. Briefly, according to the BCLC, HCC is categorized into five groups: very early stage (O), early stage (A), intermediate stage (B), advanced stage (C), as well as terminal stage (D) (Llovet et al., 2021). Of note, an analysis conducted by Richani et al. enrolled 223 HCC patients, and 5% patients were assigned in the very early stage, 35% in the early stage, 35% in the intermediate stage, 17% in the advanced stage and 18% in the terminal stage, based on the BCLC staging system (Richani et al., 2016). Generally, patients with very early-stage or early-stage HCC may benefit from resection, liver transplantation, and local alation (Llovet et al., 2016). The standard recommended treatment of patients with intermediate-stage HCC varies based on liver functions and tumor factors. Given the heterogeneity of intermediate-stage HCC, the staging system categorizes patients into 3 substages: B1, B2 and B3 (Bolondi et al., 2012). Treatment guideline recommends transplant and ablation as the treatment options for patients with stage B1. Patients with substage B2 HCC are the preferred candidates for drug-eluting bead transarterial chemoembolization (DEB-TACE) or hepatic arterial infusion chemotherapy (HAIC), while patients at substage B3 may be the candidates for systemic therapies (Kudo et al., 2015). Advanced HCC patients have very few treatment options and are basically treated with systemic therapies. Sorafenib, an oral inhibitor with the activity against vascular endothelial growth factor receptor (VEGFR) and platelet-derived growth factor receptor-β (PDGFR-β), became the standard initial treatment for advanced HCC patients (Zhang et al., 2010). Despite that nivolumab, an immunotherapy medication targeting programmed death-1 (PD1), cannot significantly prolong overall survival (OS) compared with sorafenib based on CheckMate 040 study, combination therapy of Atezolizumab (Atez) with bevacizumab (Bev) can provide clinical benefits in patients with unresectable HCC. Therefore, therapy with the combination of Atez and Bev has been utilized as a first-line treatment for advanced HCC (Finn et al., 2021). Although these therapies have substantially increased survival of HCC patients at different stages, there are still a variety of challenges ahead such as drug resistance, disease comorbidities, exorbitant costs and a lack of personalized treatment. As a result, further investigations are needed to address these issues.

## 3 m^6^A methylation regulators: writers, erasers and readers

m^6^A modification, well-recognized as the most prevalent and abundant internal RNA modification in eukaryotic RNAs, is defined as a reversible and dynamic process involving the installation or removal of a methyl (CH3) group to/from the N6 position of adenine (Chen H. et al., 2022). Previously, RNA m^6^A mainly occurs within the consensus sequence RRACH (R = A or G, H = A, C, or U), which is enriched in the stop codons and in 3′ untranslated regions of mRNA. Accumulating evidence reports the vital role of m^6^A modification in regulating RNA metabolism including RNA splicing, stability, translation, degradation and so on (He et al., 2019). In nucleus, 2 catalytic components including the methyltransferases (“writers”) and the demethylases (“erasers”) are responsible for the decoration or removal of RNA m^6^A methylation (Zaccara et al., 2019). Of note, a class of binding proteins (“readers”) can recognize m^6^A modified RNAs and therefore dictate RNA fate (Xiao et al., 2016; Shi et al., 2017). Currently, there is mounting evidence showing that m^6^A dysregulation is closely associated with the onset and development of many tumors together with aberrant expression of m^6^A regulators, including HCC (Qu et al., 2021).

### 3.1 Writers

m^6^A methylation is installed by m^6^A methyltransferases, mainly consisting of methyltransferase like-3 (METTL3) and METTL14. In addition, the newly identified m^6^A “writers” include METTL16, Wilms’ tumor 1-associating protein (WTAP), RNA binding motif protein 15 (RBM15/15B), vir-Like m^6^A methyltransferase associated (VIRMA), METTL5, and zinc-fnger CCCH-type-containing 13 (ZC3H13), Zinc Finger CCHC-Type Containing 4 (ZCCH4) (Wang et al., 2016; Ma et al., 2019).

METTL3, a predominantly enzyme of the methyltransferase complex (MTC), is responsible for the transfer of methyl groups to adenosine bases in RNA (Bokar et al., 1997). METTL14 plays an essential structural role to facilitate catalysis and recognize target RNAs though forming a stable heterodimer together with METTL3 (Wang et al., 2016). WATP, another component in the m^6^A MTC, functions to initiate and guide the localization of the METTL3-METTL14 heterodimer to the nuclear speckle (Schwartz et al., 2014). Similarly, RBM15/15B have no methyltransferase activity, but they can exert vital roles in targeting m^6^A to specific RNA sites though interaction with METTL3 and WTAP (Patil et al., 2016). VIRMA, also named as KIAA1429, mediates the preferential m^6^A methylation in the 3′-UTR and near the stop codon region of mRNAs (Yue et al., 2018). Also, it can associate with cleavage and polyadenylation specificity factor subunit 5 and 6 (CPSF5 and CPSF6) in an RNA-dependent manner, thus affecting alternative polyadenylation. METTL5, a newly identified methyltransferase containing a typical S-adenosyl-l-methionine (SAM)-binding motif, catalyzes methylation of 18S rRNA m^6^A methylation though the formation of a heterodimer with TRMT112 (van Tran et al., 2019). METTL16 emerges as a novel player in the RNA modification landscape of human cells as its significance in the addition of m^6^A methylation in U6 small nuclear RNA (snRNA) as well as the MAT2A messenger RNA (Pendleton et al., 2017). Several lines of evidence suggest the additional functions of METTL16 as it may affect mRNA splicing and stability (Mendel et al., 2018). By interacting with WTAP, ZC3H13 acts as a vital regulator of m^6^A modification as its ability to retain the writer complex in nuclear speckles (Wen et al., 2018). ZCCHC4 installs m^6^A marker in the 28S rRNAs (Ren et al., 2019). Given the fundamental role of METTL3 in m^6^A addition process, we mainly discuss the effect of METTL3 in m^6^A modification.

**FIGURE 1:**
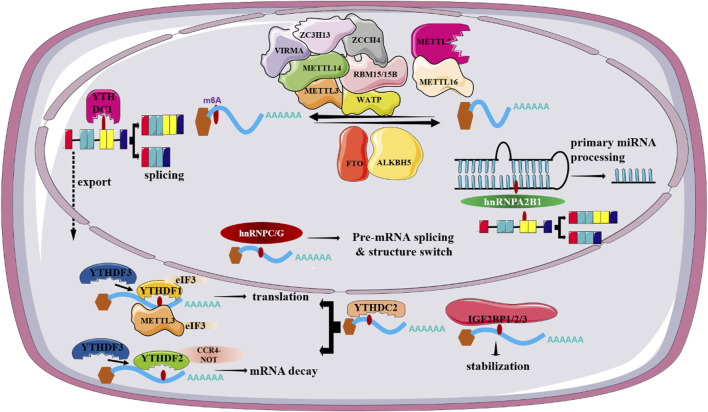
Regulatory roles of m6-related factors in RNA metabolism.

METTL3 is a major mediator of m^6^A methylation. Accordingly, knockout of METTL3 in mouse embryonic stem (ES) cells can result in near-complete loss of m^6^A modification in mRNA. Currently, multiple reports have indicated that METTL3, apart from its m^6^A methylation activities, can promote the translation of targeted RNAs, either dependent or independent on m^6^A readers. For instance, independent of m^6^A readers, METTL3 in human lung cancer can promote translation of a large subset of oncogenic mRNAs though recruiting eukaryotic translation initiation factor 3 subunit h (eIF3h) (Choe et al., 2018). Secondly, METTL3 has been documented to enhance translation by interacting with m^6^A readers. A study conducted by Wang et al. has shown METTL3 can promote maturation and activation of dendritic cell (DC) via promotion of the translation of CD80, CD40 as well as TLR4 signaling adaptor Tirap mRNA. In-depth study revealed METTL3-mediated translational enhancement of CD40 and CD80 is associated with YTHDF1 (Wang et al., 2019). Over the last decade, METTL3 has emerged as a key regulator in a variety of biological processes such as cell proliferation, cell migration and invasion, cell metabolism, and immune response via regulation of several signaling pathways including PI3K/AKT signaling, Wnt/β-catenin signaling or though shaping the epigenetic landscape. It is worthy of note that METTL3 dysregulation is closely associated with the emergence of various diseases ranging from a variety of malignant tumors to immunological and metabolic diseases (Zeng et al., 2020) (Figure 1). Particularly, there is growing evidence showing a close relationship between METTL3 loss and altered hepatocyte homeostasis and liver development defects. Barajas et al. documented that liver-specific METTL3 knockout (M3LKO) mice exhibit abnormal liver microscopic structure. Moreover, genes involved in circadian rhythm control including BMAL1 and CLOCK are dysregulated (Tang et al., 2023). Besides, hepatic Mettl3 knockout in mice can induce apoptosis and steatosis of hepatocytes, thereby eventually resulting in postnatal lethality (Xu Y. et al., 2022). Together, these studies indicated the significance of METTL3 in hepatocyte homeostasis and liver development.

### 3.2 Erasers

The m^6^A erasers, accounting for the demethylation of m^6^A in RNAs, include fat mass and obesity-associated (FTO) andα-ketoglutarate-dependent dioxygenase AlkB homolog 5 (ALKBH5) (Jia et al., 2011; Zheng et al., 2013). Despite that two enzymes are mainly localized in the nucleus, strong evidence suggests that these two proteins function independently of each other on the demethylation.

FTO is the first confirmed RNA demethylase and is expressed at high level in brain and muscle tissues. Although it was first found as an obesity-related gene (Church et al., 2010), numerous subsequent studies led to a conclusion that FTO is a key player in m^6^A RNA methylation owing to its significance in regulating mRNA processing, maturation and translation. Reports related to FTO function suggest m^6^A is the *bona fide* substrate of FTO, whereas there is emerging evidence showing FTO might not preferentially demethylate m^6^A (Mauer et al., 2017). Of note, Mauer et al. showed an obviously higher demethylation activity of FTO toward N6,2′-O-dimethyladenosine (m^6^Am) compared with that of m^6^A (Mauer et al., 2019). Despite the robust effects of FTO on m^6^Am in cells, FTO has been reported to demethylate a series of RNA modifications such as m^6^A, m^1^A, m^3^U, and m^3^T. Additionally, Zhang et al. have documented m^6^A is the most favorable substrate of FTO because the total abundance of m^6^A is at least 10-fold higher than that of m^6^Am in mRNAs (Zhang X. et al., 2019).

Notably, FTO has been reported to be frequently overexpressed in a wide range of cancer types, including breast cancer, prostate cancers, pancreatic cancer, leukemia, and so on. There is strong evidence showing that silencing of FTO can suppress tumor growth, potentiate immune-promoting response, and attenuate drug resistance, thereby highlighting the bright prospect of targeting FTO in cancer treatment (Li et al., 2022). Notably, accumulated studies have reported the vital role of FTO in hepatic lipid metabolism. Overexpression of FTO results in triglyceride accumulation in hepatocytes and AAV8-mediated FTO overexpression can promote hepatic steatosis in mice. FTO-induced lipid accumulation involves a mechanism through reducing the level of peroxisome proliferator-activated receptor α (PPARα) (Wei et al., 2022). Additionally, Tang et al. suggested the effects of FTO in modulating lipogenesis are mediated by promoting the levels of lipogenic genes including sterol regulatory element binding transcription factor 1 (SREBF1) and carbohydrate responsive element binding protein (ChREBP). Recently, Bravard et al. (2014) highlighted a novel effect of FTO in controlling leptin action via regulation of STAT3 metabolic actions in liver cells.

ALKBH5, discovered as the second m^6^A demethylase, is highly expressed in the testis and lung. Unlike FTO, ALKBH5 is found to be only demethylate m^6^A in single-stranded RNAs (ssRNAs). Accordingly, ALKBH5-deficient mice display impaired fertility resulting from abnormal expression of genes controlling spermatogenesis (Zheng et al., 2013). Apart from spermatogenesis, ALKBH5 has been reported to be involved in a wide spectrum of biological processes, including osteogenic differentiation, brain development, immune response and so on. In addition, altered ALKBH5 expression is closely associated with the onset and progression of various tumors and acts either as a tumor suppressor gene or as an oncogene, based on cancer types (Qu et al., 2022). For instance, ALKBH5 is aberrantly expressed in non-small-cell lung cancer (NSCLC) and its abnormal expression is obviously associated with unfavorable patient’s prognosis. ALKBH5-mediated reduction of RNA m^6^A levels can stabilize a variety of oncogenic drivers including UBE2C, SOX2 and MYC, thus promoting proliferation and invasion of NSCLC cells (Zhang et al., 2021). By contrast, ALKBH5 functions as a tumor suppressor in pancreatic cancer (PC), as described by a positive correlation between high ALKBH5 expression and improved survival in PC patients. Mechanically, ALKBH5 can induce PER1 expression mediated by YTHDF2, thus eventually suppressing PC progression via activation of ATM-CHK2-P53/CDC25C signaling pathway (Guo et al., 2020) (Figure 1). Specially, ALKBH5 can suppress hepatic stellate cell (HSC) activation and ameliorate liver fibrosis by triggering Patched 1 (PTCH1) activation and decreasing Dynamin-related protein 1 (Drp1) methylation in a m6A dependent fashion (Yang et al., 2022; Wang J. et al., 2023).

### 3.3 Readers

m^6^A readers, a class of regulators that function to dictate the fate of targeted RNAs by recognizing and interpreting m^6^A sites, exert a vital role in regulating RNA metabolism, including RNA splicing, export, degradation and translation. To date, m^6^A readers identified fall into 3 classes, including YT521-B homology (YTH) domain-containing proteins, heterogeneous nuclear ribonucleoproteins (HNRNPs), and insulin-like growth factor 2 mRNA-binding proteins (IGF2BPs).

The recognized YTH domain family members can be categorized into 2 subgroups, YTH domain-containing family proteins (YTHDF1/2/3, DF family) and YTH domain-containing proteins (YTHDC1/2, DC family) (Li et al., 2014). Despite the high sequence similarity, accumulating studies have shown that YTHDF1/2/3 exert different functions in gene expression. YTHDF2, as the first recognized and the most extensive studied m^6^A reader, has been documented to induce the decay of m^6^A-modified mRNAs partially though recruiting the CCR4-NOT complex via interaction with CNOT1 (Du et al., 2016). Accordingly, germ cell-specific depletion of Ythdf2 in mice results in sperm defects. Mechanically, YTHDF2 is in charge of timely clearance of m^6^A-decorated transcripts in late spermatogenesis (Qi et al., 2022). YTHDF1, on the other hand, is believed to enhance translation of m^6^A-containing transcripts by recruiting the translation initiation complex. Also, YTHDF1 facilitates ribosome loading of its targeted RNAs, further highlighting a significance of YTHDF1-assisted translation of m^6^A-modified RNAs (Wang et al., 2015). YTHDF3 not only facilitates translation through cooperation with YTHDF1 but also, in synergy with YTHDF2, mediates decay of methylated mRNA (Li et al., 2017). There is evidence that unveils the critical role of m6A readers including YTHDF1 and YTHDF2 in the progression of NAFLD. For instance, Peng et al. have reported YTHDF1 can interact with m^6^A-modified *Rubicon* transcripts and promote its stability, which in turn block the clearance of lipid droplets (Peng et al., 2022). There is strong evidence linking YTHDC1 with alternative splicing, mRNA export and chromatin modification. YTHDC1 can facilitate the binding of m^6^A-modified mRNAs to serine and arginine rich splicing factor 3 (SRSF3) and nuclear RNA export factor 1 (NXF1), thus promoting the exportation of m^6^A-containing RNAs from the nucleus to the cytoplasm (Xiao et al., 2016). Accordingly, silencing of YTHDC1 can result in accumulated methylated mRNA in the nucleus. Apart from abnormal mRNA export, deletion of YTHDC1 can lead to widespread alternative splicing defects. YTHDC2, in contrast with YTHDC1, is both nuclear and cytosolic. Kretschmer et al. has suggested that YTHDC2 can contribute to mRNA degradation by binding 5′-3′ Exoribonuclease 1 (XRN1), whereas other studies reported that it can enhance the translation of targeted mRNA in m^6^A-dependent fashion owing to containing an RNA helicase domain (Kretschmer et al., 2018).

The proteins of hnRNPs family comprise of hnRNPC, hnRNPG and hnRNPA2B1. These proteins are reported to remodel the secondary structure of targeted mRNA according to a “m^6^A-switch” mechanism, in which m^6^A induces RNA unfolding and increases the affinity of hnRNPs to ssRNA (Liu N. et al., 2015). Among them, hnRNPA2B1, acted as an RNA binding protein that exerts a vital role in regulating primary miRNA processing as well as alternative splicing, is highly expressed in a variety of human cancer types (Alarcon et al., 2015). Additionally, hnRNPC and hnRNPG are suggested to play a regulatory in mRNA splicing though processing m^6^A-containing RNA transcripts. IGF2BPs, as a novel family of m^6^A-readers, include IGF2BP1, IGF2BP2 and IGF2BP3. There is emerging evidence supporting IGF2BP proteins as RNA stabilizers in m^6^A-dependent fashion (Huang et al., 2018). However, it remains unclear whether these proteins bind to m^6^A directly as there are studies both in support of and against this idea. To date, accumulating studies document the crucial role of IGF2BPs-mediated m^6^A modification in a wide range of pathological conditions, especially cancer due to their ability to dictate mRNA fate. For instance, circular CD44 (circCD44) promotes the progression of triple-negative breast cancer (TNBC) via promotion of the stability of *Myc* mRNA though binding to IGF2BP2 (Li J. et al., 2021). Also, IGF2BPs have been reported to regulate cancer progression by interacting with m^6^A writers or erasers (Figure 1). Also, IGF2BP2 is a key regulator for hepatic outgrowth as deletion of IGF2BP2 can suppress cell proliferation (Wu et al., 2020).

## 4 The implications of m^6^A regulators in HCC progression

### 4.1 Writers

Given the crucial role of METLL3 in m^6^A methylation, the biological functions of METTL3 in the process of cancer including HCC have been widely investigated (Pan et al., 2021). To date, accumulating studies have reported that METTL3 can serve as an oncogene in HCC progression though various mechanisms. *In vivo* studies found that METTL3 can contribute to HCC tumorigenicity and lung metastasis (Chen et al., 2018). In consistent, METTL3 not only increases the proliferation, migration and invasion of HCC cells but also promotes glycolysis and lipogenesis to facilitate HCC progression (Lin Y. et al., 2020; Zuo et al., 2020). Mechanically, Chen et al. have documented METTL3 can promote HCC progression though activating the Janus kinase/signal transducers and activators of transcription (JAK/STAT) pathway. In-depth investigations revealed that the promoting effect of METTL3 on the JAK/STAT pathway is mediated by suppressing Suppressor of Cytokine Signaling 2 (SOCS2), a suppressor of the JAK/STAT pathway, in an m^6^A-YTHDH2-dependent fashion (Chen et al., 2018). Also, METTL3 has been demonstrated to inhibit RAD52 Motif Containing 1 (RDM1) mRNA expression in an m^6^A-dependent manner, thereby ultimately promoting the growth of HCC cells via repression of p53 signaling pathway (Chen S. L. et al., 2020). Li et al. suggested ubiquitin specific peptidase 7 (USP7) accounts for the oncogenic role of METTL3 (Li Y. et al., 2021). Recently, Chen et al. documented BMI1 and RNF2, two crucial elements of the polycomb repressive complex 1 (PRC1), are direct targets of METTL3. Deletion of YTHDF1 remarkably decreases the expression of BMI1 and RNF2, thereby showing METTL3 facilitates HCC progression though m^6^A methylation of BMI1 and RNF2 in a YTHDF1-dependent mechanism (Chen W. et al., 2022). Besides, METTL3 serves as a promoting element in the epithelial-mesenchymal transition (EMT) process in HCC by enhancing the translation of Snail mRNA via an m^6^A-YTHDF1 fashion (Lin et al., 2019). Additionally, several mechanisms concerning the regulatory role of METTL3 in metabolic rewiring of HCC have been discovered. For instance, overexpressing METTL3 has been reported to induce glycolysis of HCC cells via induction of hepatitis B X-interacting protein (HBXIP) expression, enhancement of hypoxia-inducible factor-1 alpha (HIF-1α) level, as well as activation of mTORC1 signaling pathway (Lin Y. et al., 2020; Yang et al., 2021). Moreover, high METTL3 expression increases the stability of long intergenic non-protein coding RNA 958 (LINC00958) and promotes its expression, thus eventually contributing to a more activated lipogenic phenotype by increasing HDGF expression via inhibition of the interaction between miR-3619-5p and HDGF (Zuo et al., 2020). Clinically, patients with a relatively low METTL3 expression exhibit a favorable prognosis than those with high METTL3 expression. Taken together, METTL3 may serve as a promising therapeutic target for HCC treatment. Apart from the HCC-promoting effect, METTL3 has been reported to be closely associated with sorafenib resistance in chemotherapy of advanced HCC patients, described as a reduced expression of METTL3 in sorafenib-resistance HCC. Mechanistically, METTL3 deletion decreases the transcription efficiency of Forkhead box O3 (FOXO3) via a YTHDF1-dependent fashion and thereby promotes autophagy, a crucial process in multidrug resistance in chemotherapy of cancer, thus ultimately resulting in sorafenib resistance (Lin Z. et al., 2020). Given the crucial roles of METTL3 in the progression and drug resistance in HCC, more efforts are required to disclose its functions and the relevant mechanisms in HCC. In contrast, the roles of METTL14 in HCC are controversial. For instance, Ma et al., suggested METTL14 serves as a tumor suppressor in HCC progression. Moreover, METTL14 is remarkably decreased in HCC tissues and acts as a prognostic factor for tumor recurrence in HCC. Consistently, METTL14 suppresses HCC metastasis though enhancing the recognition of pri-miR126 by DGCR8, thus eventually increasing the expression of miR-126 (Ma et al., 2017). Also, Shi et al. reported METTL14 inhibits the invasion of HCC via modulation of the EGFR/PI3K/AKT signaling pathway (Shi Y. et al., 2020). However, Chen et al., suggested the expression of METTL14 is not obviously reduced in HCC (Chen et al., 2018). Of note, they also found a tumor promoting effect of METTL14 in the proliferation and migration of liver cancer cells. Owing to the contradictory findings of METTL14 in previous studies of HCC, Zhang et al., analyzed HCC tissues and paired adjacent samples in multiple microarray datasets. Although the reasons for these contradictions remain as an open question, they indicated the paradoxical expression patterns of METTL14 in HCC samples may be ascribed to the heterogeneity of HCC samples. In addition, the difference in HCC cells, the versatility of METTL3-METTL14 heterocomplex, as well as m^6^A-independent manner might lead to the contradictory results of the functions of METTL14 on HCC metastasis (Zhang B. H. et al., 2019).

**FIGURE 2:**
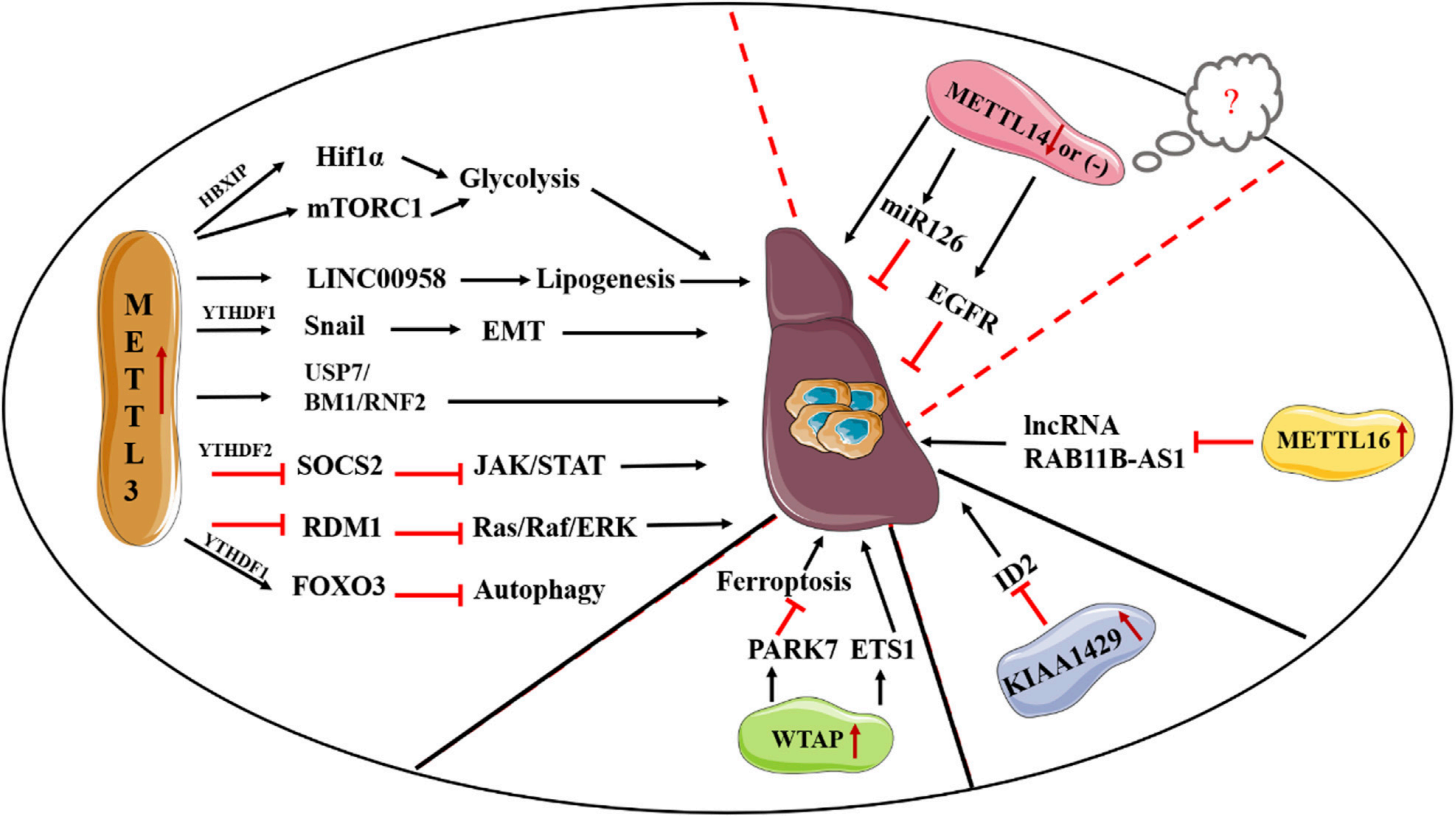
Deregulation of “writers” in HCC.

WTAP is highly expressed in HCC samples and is closely associated with unfavorable prognosis of HCC. Similarly, Duan et al., found that patients with a relatively high WTAP expression suffer worse recurrence-free survival (RFS) in HBV-positive Asian small HCC patients (Duan et al., 2022). In consistent, both *in vitro* and *in vivo* studies supported an oncogenic role of WTAP in HCC progression. Mechanistically, deletion of WTAP can result in G2/M arrest in HCC in a p21/p27-dependent fashion with the involvement of ETS proto-oncogene 1 (ETS1) (Chen et al., 2019). Second, Chen et al. reported WTAP promotes HCC carcinogenesis by altering the m^6^A methylation of CircCMTM3, thus suppressing HCC ferroptosis (Chen S. et al., 2022). KIAA1429 is also highly expressed in HCC tissues, and high expression of KIAA1429 indicates the poor outcome of HCC patients. First, Cheng et al. suggested that the oncogenic role of KIAA1429 is mediated by inhibiting inhibitor of DNA binding 2 (ID2) though upregulating m^6^A-modified ID2 transcripts (Cheng X. et al., 2019). Second, Liu et al. found that KIAA1429 could affect pre-mRNA splicing of cancer-associated genes. Third, KIAA1429 promotes EMT process in sorafenib-resistant HCC in an m^6^A-dependent manner (Kuang et al., 2023). METTL16 is elevated in HCC tissues and high expression of METTL16 is closely associated with unfavorable prognosis of HCC patients. In consistent, METTL16 can serve as a promoter in the growth and metastasis of HCC. In-depth exploration indicated METTL16 promotes HCC progression via downregulation of lncRNA RAB11B-AS1 though inducing m^6^A methylation (Dai et al., 2022) (Figure 2).

### 4.2 Erasers

To date, the expression and regulatory roles of FTO in HCC are perplexing. Ma et al. reported an obviously decreased expression of FTO in HCC tissues (Ma et al., 2017), whereas Li et al. (2019) suggested that FTO is highly expressed in HCC tissues and cells and can serve as a prognostic marker in HCC individuals. In addition, they suggested that FTO can promote HCC carcinogenesis by inducing the demethylation of pyruvate kinase M2 (PKM2), a key enzyme of glycolysis. In contrast, Liu et al. documented that silent information regulator 1 (SIRT1) acts as an oncogene by inhibiting FTO expression via RANBP2-mediated SUMOylation. Furthermore, silencing of FTO increases the m^6^A-modified Guanine nucleotide-binding protein G (o) subunit alpha (GNAO1) transcripts and thereby downregulates its expression, thus promoting HCC progression (Liu X. et al., 2020). Similarly, Mittenbuhler et al. (2020) showed a protective role of FTO in chemically-induced HCC tumorigenesis and the tumor-suppressing effect of FTO might be ascribed to the decreased CUL4A protein expression. Third, Liu et al. also proposed FTO serves as a tumor suppressor in HCC development, described as a dramatically decreased FTO expression in HCC tissues and a significant lower in cell proliferation and invasion capability following overexpression of FTO. ALKBH5 is dramatically decreased in HCC tissue samples and a lower ALKBH5 expression implies poor outcomes of HCC patients (Chen Y. et al., 2020). In consistent, high expression of ALKBH5 can restrain HCC metastasis, thus suggesting a tumor-suppressive function of ALKBH5 in HCC progression. Additionally, LINC02551 is negatively regulated by ALKBH5 in an m^6^A-dependent fashion. Functionally, LINC02551 acts as a tumor-promoting factor though suppressing the interaction between DDX24 and a E3 ligase TRIM27, thus promoting degradation of DDX24 (Zhang et al., 2022). Lastly, Wang et al. revealed ALKBH5 can reduce progestin and adipoQ receptor 4 (PAQR4) expression in an m^6^A-IGF2BP1 dependent manner, thereby suppressing the PI3K/AKT pathway activity and ultimately promoting HCC progression (Wang W. et al., 2023). Of note, cIARS positively regulates sorafenib-induced autophagy and ferritinophagy via repression of the ALKBH5-mediated autophagy inhibition (Liu Z. et al., 2020).

### 4.3 Readers

Accumulating studies display strong evidence linking m^6^A readers with HCC tumorigenesis. YTHDF1 is highly expressed in HCC tissue samples and closely correlated with HCC grade (Zhao et al., 2018). Similarly, YTHDF1 serves as an oncogene in HCC progression, described as a decreased HCC cell proliferation and metastasis resulting from deletion of YTHDF1. Functionally, YTHDF1 can activate the PI3K/AKT/mTOR signaling pathway, thereby contributing to HCC progression (Luo et al., 2021). Additionally, Wang et al. found HIF-1α can promote YTHDF1 transcription under hypoxic conditions. In-depth exploration revealed that YTHDF1 facilitates autophagy-related malignancy of HCC though promoting translation of ATG2A and ATG14 in an m^6^A-dependent fashion (Li Q. et al., 2021). Currently, the regulatory roles of YTHDF2 are contradictory. For instance, Zhang et al. documented that loss of YTHDF2 can impair the liver cancer stem cell (CSC) phenotype and inhibit cancer metastasis though decreasing the m^6^A methylation of OCT4 transcript, thereby showing YTHDF2 can act as a major oncogene driver of HCC (Zhang et al., 2020). Moreover, YTHDF2 O-GlcNAcylation is reported to be obviously upregulated in HBV-associated HCC tissues. O-GlcNAcylation of YTHDF2 facilitates HBV-related HCC progression via promotion of the stability of MCM2 and MCM5 transcripts (Yang et al., 2023). In contrast, YTHDF2 might serve as a tumor suppressor in HCC development, as two studies provided evidence for hypoxia-mediated YTHDF2 reduction. The former study showed YTHDF2 can suppress cell proliferation via promotion of the degradation of epidermal growth factor receptor (EGFR) mRNA in HCC (Zhong et al., 2019). In addition, Hou et al. found that deletion of YTHDF2 can fuel inflammation and vascular reconstruction. Functionally, YTHDF2 destabilizes m^6^A-modified interleukin 11 (IL11) and serpin family E member 2 (SERPINE2) mRNAs, which contributes to the inflammation-mediated malignancy (Hou et al., 2019). Zhou et al. and Guo et al. suggested a tumor-promoting effect of YTHDF3 in HCC. The former study revealed a dramatically increased YTHDF3 expression in HCC tissue samples and loss of YTHDF3 can lead to a decrease of the growth and metastasis of HCC by inducing phosphofructokinase PFKL expression in an m^6^A-dependent fashion (Zhou et al., 2022). Besides, lysine-specific demethylase 5B (KDM5B) facilitates HCC progress via modulation of miR-448/YTHDF3/ITGA6 axis (Guo et al., 2021). YTHDC1 is remarkably overexpressed in HCC tissue samples. In accordance, high expression of YTHDC1 indicates a poor survival of HCC patients. Mechanistically, YTHDC1 can favor the cytoplasmic output of m^6^A-modified circHPS5, which can serve as a miR-370 sponge to modulate HMGA2 expression, thereby accelerating HCC tumorigenesis (Rong et al., 2021). Furthermore, YTHDC1 facilitates the back splicing and biogenesis of circ-ARL3 in an m^6^A-dependent fashion, which in turn promotes HBV-associated HCC progression by sponging miR-1305 (Rao et al., 2021).

There is strong evidence linking IGFBPs with HCC carcinogenesis (Lin et al., 2021). To date, the expression pattern of IGFBP-1 in HCC specimens is controversial, showing either a higher expression of IGFBP-1 in HCC tissues by Gutschner et al. or a decreased mRNA level of IGFBP-1 in HCC specimens. In line with contradictory expression pattern, studies concerning the role of IGFBP-1 in regulating malignant behaviors of HCC have also yielded contrasting results. There is evidence providing that the oncogenic effect of IGFBP-1 is ascribed to its mRNA processing capabilities as it can stabilize c-Myc transcripts (Huang et al., 2018). Also, IGF2BP1 induces SRF expression via an m^6^A-dependent manner, thus favoring HCC cell proliferation and invasion (Muller et al., 2019). Conversely, IGF2BP1 has been documented to decrease the potential of HCC cells to induce lymphangiogenesis (Geis et al., 2015). IGF2BP2 is overexpressed in HCC tissues and is positively associated with worse histological grade of HCC. Functionally, Liu et al. reported downregulated AKT and ERK pathways contribute to miR-216b-mediated suppression of HCC tumorigenesis (Liu F. Y. et al., 2015). Of note, studies regarding the regulatory roles of IGF2BP3 in HCC development have yielded inconsistent conclusions. Firstly, Nguyen et al. (2014) demonstrated that LIN28B preferentially requires IGF2BP3 to perform tumor-promoting effects. Besides, Gao et al. (2020) suggested IGF2BP3 can enhance miR191-5p-mediated inhibition of ZO-1 signaling, thus acting as a driver of malignancy of HCC. Furthermore, loss of IGF2BP3 obviously induces ferroptosis in HCC cells by decreasing NRF2 mRNA stability in an m^6^A-dependent manner after sorafenib treatment (Lu et al., 2022). Conversely, another study showed that IGFBP-3 suppresses HCC cell proliferation via inhibition of basic fibroblast growth factor (bFGF) and platelet-derived growth factor (PDGF) expression (Ma et al., 2016).

Recently, accumulating literature indicated hnRNPC can serve as an oncogene in HCC progression, described as an elevated expression of hnRNPC in HCC tissues and a decrease of tumor growth and metastasis following hnRNPC silencing. Mechanistically, hnRNPC may exert a tumor-promoting effect via mechanisms involving suppression of the Ras/MAPK signaling pathway or IL-6/STAT3 signaling, or reduction of HIF-1α expression (Hu et al., 2021; Liu D. et al., 2022). Regarding hnRNPA2B1, trichostatin A (TSA)-induced lncRNA-uc002mbe.2 can directly bind to hnRNPA2B1 in Huh7 cells, which in turn deactivates ATK activity and promotes p21 expression, thus ultimately suppressing HCC progression (Chen et al., 2017). Additionally, miR503HG serves as a tumor suppressor in HCC progression by decreasing hnRNPA2B1 expression via a ubiquitin-proteasome pathway, thus eventually suppressing NF-κB signaling pathway (Wang et al., 2018).

## 5 The implications of m^6^A regulators in ICC progression

The regulatory roles of m^6^A regulators in ICC have rarely been investigated. To date, Xu et al. reported an elevated expression of METTL3 in ICC tissues and high METTL3 expression indicates an unfavorable survival in ICC patients. METTL3-driven IFIT2 mRNA degradation in a YTHDF2-dependent fashion is demonstrated to facilitate ICC progression (Xu Q. C. et al., 2022). Additionally, METTL3 can upregulate hepatic leukemia factor (HLF) expression in an m^6^A-dependent manner. HLF accelerates tumor growth and metastasis via modulation of frizzled-4 (FZD4) and forkhead box Q1 (FOXQ1). Meanwhile, FOXQ1 transcriptionally activates METTL3 expression, which in turn activates WNT/β-catenin signaling, thereby ultimately promoting ICC progression (Xiang et al., 2023). VIRMA, as another m^6^A writer, is closely associated with adverse prognosis of ICC patients and promotes proliferation and metastasis of ICC though inducing SIRT1 expression via a mechanism involving m^6^A modification (Zhou et al., 2023). Regrading m^6^A erasers, FTO is downregulated in ICC tissue specimens and a higher expression of FTO predicts favorable prognosis in ICC patients. Moreover, loss of FTO can promote anchorage-independent growth and mobility of ICC cells via destabilization of TEAD2 mRNA (Rong et al., 2019). Lastly, Huang et al. (2022) suggested YTHDF1 serves as an oncogene in ICC progression though regulating the translation of EGFR mRNA via an m^6^A-dependent manner. Taken together, more efforts are required to further illustrate the regulatory effects of m^6^A regulators in ICC.

## 6 The implications of m^6^A regulators in hepatoblastoma progression

HB, originated from undifferentiated hepatic progenitor cells, is the most common type of liver cancer in children. Currently, Liu et al. reported an increase of m^6^A methylation in HB. Moreover, METTL3-induced altered methylation can activate the Wnt/β-catenin signaling pathway, thus promoting CTNNB1 expression and eventually facilitating HB tumorigenesis (Liu et al., 2019). Solute carrier family 7 member 11 (SLC7A11) exerts a tumor-promoting effect via inhibition of ferroptosis. METTL3-mediated m^6^A methylation can stabilize SLC7A11 mRNA and promote its expression via an IGF2BP1-dependent fashion (Liu L. et al., 2022). In consistent, loss of METTL3 can enhance the sensitivity of HB cells to ferroptosis. In summary, the regulatory roles of m^6^A modification in HB tumorigenesis are needed further studies.

## 7 Therapeutic applications of m^6^A regulators in liver cancer

Owing to the vital roles of m^6^A RNA methylation in modulating liver cancer progression, manipulating RNA methylation may be a promising therapeutic approach for the treatment of liver cancer. To date, m^6^A regulator-based signature for predicting prognosis in patients with HCC has been documented in a variety of studies. A set of m^6^A regulators, including METTL3, YTHDF1, YTHDF2, IGFBP1, IGFBP3, WTAP, and so on, are considered as unfavorable prognostic indicators. Meanwhile, ZC3H13 is deemed a favorable prognostic factor. Moreover, there is growing evidence showing a close relationship between m^6^A RNA methylation and the abundance of infiltrating immune cells (Xu et al., 2021). Han et al. (2019) reported Ythdf1-deficient mice exhibit an enhanced antigen-specific CD8^+^ T cell anticancer response. Also, the loss of YTHDF1 can improve the therapeutic effectiveness of PD-L1 checkpoint blockade, thus indicating YTHDF1 might be a promising therapeutic target in immunotherapy. Notably, ZC3H13 expression is reported to be positively associated with infiltrating immune cells, thereby facilitating the elimination of HCC cells and eventually improving prognosis (Xu et al., 2021).

Additionally, a considerable progress has been made concerning the development of specific inhibitors that target m^6^A regulators. Given the vital roles of FTO in tumorigenesis and drug resistance, developing specific inhibitors targeting FTO has attracted much attention. First, rhein, a natural compound that extracted from herbal plants, is reported to inhibit FTO activity and increase cellular m^6^A levels by competitively binding to the active site of FTO. However, it is not an FTO-specific inhibitor because it also targets ALKBH5 (Chen et al., 2012). Second, Meclofenamic acid (MA), a widely used anti-inflammatory drug, can dramatically increase cellular m^6^A levels by inhibiting FTO. Yan et al. (2018) suggested MA can override tyrosine kinase inhibitor (TKI) resistance. Based on a structure-guided method, two novel FTO inhibitors, FB23 and FB23-2, were developed. Huang et al. (2019) reported an obviously tumor-suppressing effect of FB23-2 in acute myeloid leukemia (AML) cells and FB23-2 treatment can improve the survival of leukemic mice. Moreover, FB23-2 exhibits a safe toxicity profile in in vivo studies. Notably, owing to the strong evidence showing a close relationship between METTL3 and the initiation and development of multiple cancers, targeting METTL3 might be a promising avenue for cancer treatment. STM2457, a first-in-class catalytic inhibitor of METTL3, can result in a decrease in AML growth and an increase in cellular apoptosis. Mechanistic studies suggested the tumor-suppressing effect of STM2457 is driven by selectively reducing m^6^A levels on several leukaemogenic mRNAs (Yankova et al., 2021). Therapeutic resistance, a severe obstacle in the field of cancer treatment, can lead to cancer recurrence and progression. As a result, it is of significance to investigate the potential therapeutic approaches to target cancer treatment resistance. Increasing evidence suggests m^6^A regulators play critical role in regulating therapeutic resistance via multiple mechanisms including promotion of DNA damage repair, modulation of metabolic rewiring, remodeling tumor microenvironment (TME), and so on (Wang D. et al., 2023). Enforced hepatocyte nuclear factor 3γ (HNF3γ) expression can sensitize HCC cells to sorafenib-induced cell apoptosis by promoting OATP1B1 and OATP1B3 expression. Moreover, METTL14 is involved in the HNF3γ reduction in HCC cells, thereby highlighting the clinical potential of m^6^A regulators in reversing drug resistance (Zhou et al., 2020). Immunotherapy, referred to treatments that exert anti-tumor activities though suppressing immunosuppressive factors including PD-1 or its ligand PD-L1, has shown excellent clinical results in various types of cancer. However, a significant proportion of cancer patients has no response to immunotherapy, therefore, the clinical application of immunotherapy is limited. Currently, accumulating studies documented m^6^A regulators as significant factors in remodeling TME, thus affecting the treatment response to immunotherapy. Li et al. have reported a critical role of ALKBH5 in controlling the efficacy of immunotherapy as deletion of ALKBH5 changes metabolite contents including lactate in the TME, which can alter immune cell infiltration. Moreover, ALK-04, a small-molecule inhibitor of ALKBH5, enhances immunotherapy outcomes, thereby suggesting that combinatorial therapy with ALKBH5 inhibitors might be an approach to overcome the resistance for immunotherapy (Li et al., 2020). Although several inhibitors targeting FTO or METTL3 are reported, there are quite a few inhibitors targeting other m^6^A regulators, thus deserving further exploration. Moreover, none of these reported inhibitors have been approved for clinical use. As a result, a series of preclinical and clinical trials should be carried out to investigate safety profiles, therapeutic effectiveness, as well as pharmacokinetics to facilitate the clinical use of inhibitors targeting m^6^A factors. Additionally, the efficacy of inhibitors targeting m^6^A regulators in combination with other therapies should be further investigated.

## 8 Conclusion and perspectives

Advances in RNA-sequencing technologies, including single-cell RNA sequencing, facilitate the new understanding of m^6^A RNA methylation not only of its effects in various diseases but also its potential therapeutic implications. Abundant studies support the essential role of m^6^A RNA methylation in liver tumorigenesis (Ding et al., 2024). Our review provides an overview of regulatory roles and mechanisms of m^6^A RNA modification in liver carcinogenesis, showing m^6^A methylation regulators are frequently aberrantly expressed in liver cancer tissues and are involved in the initiation and progression of liver cancer though various mechanisms including regulation of cell cycle, apoptosis, promotion of cellular metabolism, as well as modulation of TME. Moreover, we also discuss the clinical applications, in hope of providing novel therapeutic strategies for the treatment of liver cancer by targeting the m^6^A machinery. Overall, considerable and valuable insights have been gained from m^6^A studies in the field of liver cancer. Yet, the illustrations of m^6^A dysregulation in liver cancer remain vastly unexplored, especially in ICC and HB. Of note, several studies have yielded contradictory results on the changes of the expression levels of m^6^A factors as well as their functions. These paradoxical results might be ascribed to the heterogeneity of liver cancer, small sample sizes, the difference in cell background. Therefore, in-depth explorations are needed to ensure the validity of our understanding of m^6^A methylation in liver cancer progression. It may be useful to address these issues though establishing consensus guidelines for sequencing and analysis methodologies, as well as constructing standard cell/animal models. Additionally, increasing evidence suggests m^6^A regulators may exert similar functional effects in the initiation and development of liver cancer though regulating distinct sets of genes or the same set of genes via different mechanisms, therefore, the modification specificity of m^6^A regulators is needed to be clarified.

Despite great progress, there are still a variety of questions that warrant in-depth investigation: 1) reconcile above-mentioned contradictory findings to further illustrate the underlying mechanisms; 2) screen blood-based m^6^A-related diagnostic and prognostic biomarkers for liver cancer; 3) develop novel specific inhibitors for m^6^A regulators and clarify their pharmacokinetics and safety profiles; 4) investigate dynamics of m^6^A RNA methylation during liver carcinogenic process; 5) elucidate context-specific m^6^A functions in different subtypes of liver cancer. 6) explore the efficacy of therapies that combine m^6^A-targeting inhibitors and commonly utilized immune checkpoint blockades or other existing anti-tumor approaches. In a word, addressing these limitations is beneficial for deepening our understanding of the m^6^A involvement in liver cancer progression and the advances of novel therapeutic approaches to improve the life quality of HCC patients.
